# Demonstration of Enhanced Piezo-Catalysis for Hydrogen Generation and Water Treatment at the Ferroelectric Curie Temperature

**DOI:** 10.1016/j.isci.2020.101095

**Published:** 2020-04-24

**Authors:** Pham Thi, Thuy Phuong, Yan, Zhang, Nick, Gathercole, Hamideh, Khanbareh, Nguyen Phuc, Hoang Duy, Xuefan, Zhou, Dou, Zhang, Kechao, Zhou, Steve, Dunn, Chris, Bowen

**Affiliations:** 1 State Key Laboratory of Powder Metallurgy, Central South University, Changsha, Hunan, 410083, China; 2 Institute of Chemical Technology, Viet Nam Academy of Science and Technology, Ho Chi Minh, Vietnam; 3 Department of Mechanical Engineering, University of Bath, Bath BA2 7AY, UK; 4 Chemical Engineering, School of Engineering, London South Bank University, 103 Borough Road, London SE1 0AA, UK

**Keywords:** Catalysis, Electrochemical Energy Production, Ceramics

## Abstract

Hydrogen can contribute significantly to the energy mix of the near future, as it is an attractive replacement for fossil fuels due to its high energy density and low greenhouse gas emission. A fascinating approach is to use the polarization change of a ferroelectric due to an applied stress or temperature change to achieve piezo- or pyro-catalysis for both H_2_ generation and wastewater treatment. We exploit low Curie temperature (*T*_*c*_) ferroelectrics for polarization-driven electrochemical reactions, where the large changes in polarization and high activity of a ferroelectric near its *T*_*c*_ provides a novel avenue for such materials. We present experimental evidence for enhanced water splitting and rhodamine B degradation via piezo-catalysis by ultrasonic excitation at its *T*_*c*_. Such work provides an effective strategy for water splitting/treatment systems that employ low *T*_*c*_ ferroelectrics under the action of mechanical stress or/and thermal fluctuations.

## Highlights

• First demonstration of the positive impact of operating near *T*_*c*_ for piezo-catalysis
• Ultrasound applied to achieve piezo-catalysis near *T*_*c*_
• High hydrogen production rate and degradation rate were achieved at the *T*_*c*_
• Piezo-catalysis is a new avenue for low *T*_*c*_ and lead-free ferroelectrics

## Graphical Abstract

**Figure:**
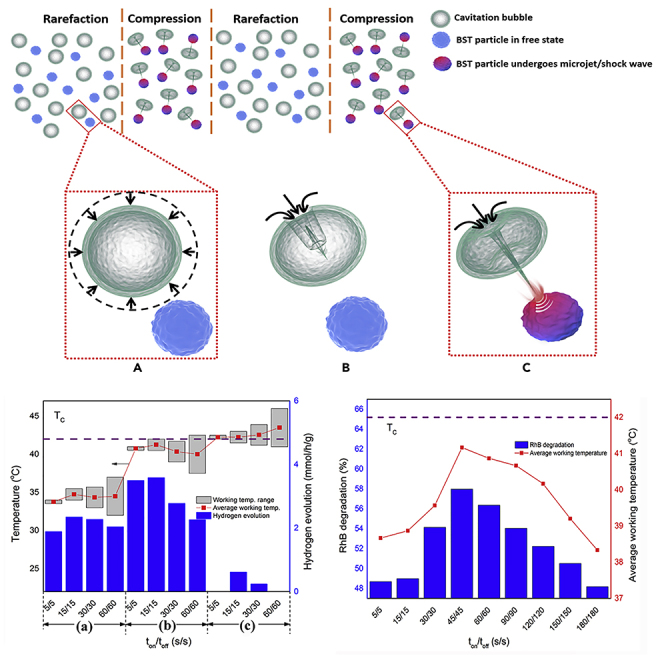

## Introduction

Hydrogen is considered as a promising and abundantly available renewable fuel with a high energy density per unit mass, with potential to act as a carbon-free energy carrier in response to the global energy crisis and need to reduce environmental pollution (Xiang et al., 2016, Zhu et al., 2018). The ability to couple piezoelectric and pyroelectric effects with electrochemical processes continues to attract attention to exploit mechanical vibrations and temperature fluctuations for hydrogen generation, water treatment, photocatalysis, and materials processing (Zhang et al., 2015, Zhang et al., 2017, Ismail et al., 2019, Mushtaq et al., 2018). Starr et al. (Starr et al., 2012, Starr and Wang, 2013, Starr and Wang, 2015) provided a detailed overview of the fundamentals of piezo-catalysis and examined the surface electrochemistry of a piezoelectric whose polarization changed in response to a mechanical strain. To demonstrate its potential, hydrogen generation was achieved by oscillating a piezoelectric Pb(Mg_1/3_Nb_2/3_)O_3_-32PbTiO_3_ (PMN-PT) cantilever in contact with deionized water (Starr and Wang, 2013). Piezoelectric materials that have been examined include zinc oxide, which have been combined with photocatalytic studies by the application of both ultrasound and light (Xue et al., 2015, Tan et al., 2015, Ma et al., 2019, Hong et al., 2016), or heat to explore pyroelectric effects due to a change in polarization with temperature (Qian et al., 2017, Tang et al., 2018). Single- and few-layer piezoelectric materials, such as MoS_2_ and MoSe_2_, have also been examined in detail by Wu *et al.* (Wu et al., 2016b, Wu et al., 2017, Wu et al., 2018c, Lin et al., 2017).

Recent work by Kakekhani et al. (Kakekhani and Ismail-Beigi, 2015, Kakekhani and Ismail-Beigi, 2016a, Kakekhani and Ismail-Beigi, 2016b, Kakekhani et al., 2016) has focused on the use of ferroelectric materials for polarization-driven catalysis, which exhibit both piezo- and pyroelectricity. These materials are of interest because their polarization direction can be changed, thereby enabling a switching of surface chemistry. Modeling work, based on PbTiO_3_, showed that splitting of water into oxygen and hydrogen is possible by thermally cycling a ferroelectric above and below its Curie temperature (*T*_*c*_) when in contact with water. Initial experimental effort to demonstrate this process used pyroelectrics as an external charge source to generate a potential difference for water splitting (Zhang et al., 2019, Xie et al., 2017). However, recent work has shown that it is possible to generate hydrogen by simply placing ferroelectric particles in direct contact with water and subjecting them to cyclic heating and cooling (Belitz et al., 2017, Xu et al., 2018b, You et al., 2018a). As a model ferroelectric material, barium titanate has often been explored for tribo- (Li et al., 2019), piezo- (Belitz et al., 2017, Feng et al., 2019, Hong et al., 2010, Hong et al., 2012, Jin et al., 2019, Pan et al., 2019, Wu et al., 2018a), and pyroelectric catalysis (Benke et al., 2015, Wu et al., 2018b, Xia et al., 2017). This is primarily due to its lead-free nature, low-cost, and ease of manufacture in a wide variety of forms, such as nanofibers (Wu et al., 2018a, Xia et al., 2017), composites (Min et al., 2018), and particles (Belitz et al., 2017), which can be decorated with semiconductors (Liu et al., 2019) to enhance catalytic performance.

The working temperature of a ferroelectric, in relation to its Curie temperature, is likely to strongly influence any polarization-driven catalysis because the piezoelectric, pyroelectric (Zhang et al., 2020), dielectric, and electrocaloric properties are often maximized near the Curie temperature due to (1) intrinsic effects as a result of the large changes in polarization with stress or temperature or (2) the enhanced extrinsic contributions associated with domain wall motion (Karthik and Martin, 2011). However, it is surprising that there has been little effort to date that examines this experimentally. Evidence of a ferroelectric enhancement in a photochemical environment has been reported (Cui et al., 2013, Cui et al., 2019), where the photocatalytic degradation of a dye molecule was enhanced below the *T*_*c*_ due to an enhanced carrier lifetime (Morris et al., 2016).

Although barium titanate has been widely explored for polarization-driven catalysis, its Curie temperature is above the boiling point of water (*T*_*c*_ ∼ 120°C (Harwood et al., 1947)), thereby making an evaluation of catalytic performance in water above and below *T*_*c*_ impossible. Other high Curie temperature ferroelectrics studied to date with a *T*_*c*_> 100°C include LiNbO_3_ (Gutmann et al., 2012), LiTaO_3_ (Gutmann et al., 2012), PbZr_x_Ti_1-x_O_3_ (Lin et al., 2014, Feng et al., 2017), BiFeO_3_ (Liu and Wu, 2019, Wu et al., 2016a, You et al., 2019), NaNbO_3_ (You et al., 2018b, Wang et al., 2019) (*T*_*c*_ ∼ 350°C (Guo et al., 2015)), ZnSnO_3_ (Wang and Wu, 2020), and KNbO_3_ (Jia et al., 2019) (*T*_*c*_ *∼* 434°C (Matthias and Remeika, 1951)), where piezo-catalytic hydrogen generation and degradation of water contaminants has been achieved at temperatures that are well below *T*_*c*_. One low Curie temperature material (*T*_*c*_< 100°C) that has been studied is barium strontium titanate (Xu et al., 2018a, Xu et al., 2018b), although the influence of operating temperature on piezo- or pyro-catalytic performance has yet to be been examined.

In this paper we provide the first examination of the influence of working temperature, in relation to the *T*_*c*_, on the *piezo-catalytic* performance for hydrogen generation and degradation of water contaminants (rhodamine B [RhB]) under the application of ultrasound. We have selected a BaTiO_3_-based solid solution based on Ba_0.75_Sr_0.25_TiO_3_ due to its relatively low Curie temperature (*T*_*c*_ ∼ 42°C), which allows us to systematically examine piezo-catalysis at temperatures (1) below *T*_*c*_, (2) at the *T*_*c*_, and (3) above *T*_*c*_. Additional advantages include its lead-free composition, low cost, and comparable ferroelectric, piezo-, and pyroelectric performance with respect to lead-based piezoelectric ceramics (Wu, 2018, Airimioaei et al., 2017).

## Results and Discussion

Although the initial pioneering work of Starr et al. (Starr and Wang, 2013) used a simple cantilever configuration to generate a strain in the piezoelectric, a number of researchers have used ultrasound to apply a mechanical deformation. A difference between these two approaches is that the application of ultrasound can lead to the formation of bubbles by cavitation of water, whose subsequent violent collapse can create localized hotspots and high pressures to generate temperatures of ∼5000°C and pressures of ∼500 atm. Such cavitation events have also been shown to generate hydrogen from water (Islam et al., 2019), termed *sono-chemical* generation in this paper. As a result, close control of operating temperature during the application of ultrasound was undertaken in this work, along with the use of control samples that contained non-ferroelectric ceramic particles (termed *control*) or were particulate free (termed *blank*) to separate the sono-chemical and piezo-catalytic effects, a factor that is often ignored. By careful experimental design, the results presented here therefore provide new insights into the influence of operating temperature, in relation to the *T*_*c*_, on piezo-catalytic performance and demonstrates the importance of selecting an optimum operating temperature or *T*_*c*_ for improved water splitting or water treatment; it also demonstrates that harnessing the inherent polarization changes in ferroelectrics with stress and temperature can aid chemical conversion.

**Figure 1:**
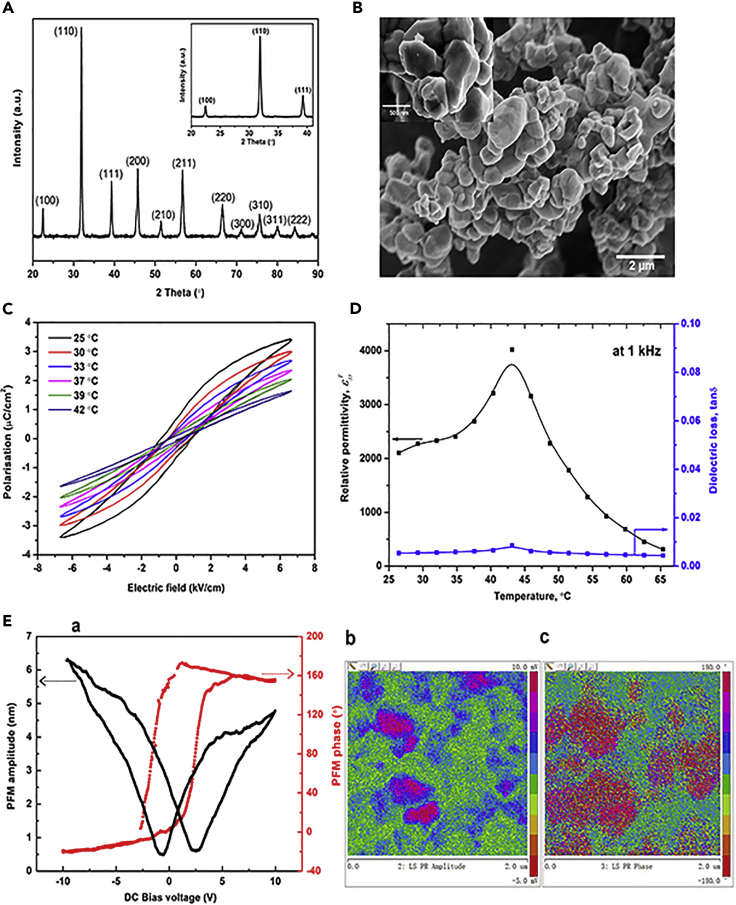
X-ray Diffraction (XRD) Patterns, Microstructural Morphology, Dielectric and Ferroelectric Properties of Synthesized BST (A) XRD patterns of the synthesized powder. (B) SEM image of the synthesized BST powder, inset shows a higher magnification. (C) polarization-electric field (*P-E*) loops of sintered BST as a function of temperature and electric field. (D) relative permittivity and dielectric loss of the sintered BST dense pellet as a function of temperature at a frequency of 1 kHz. (E) local ferroelectric domain switching. (a) Local hysteresis loop behavior for the amplitude and phase, (b) PFM amplitude, (c) PFM phase.

In terms of the ferroelectric Ba_x_Sr_1-x_TiO_3_ (BST) material used for this study, Figure 1A shows the XRD data of the synthesized powders with Miller indices indicated in the 2θ range of 20–90°, where the sharp and well-defined XRD peaks indicate its high crystallinity. Only one crystalline (inset of Figure 1A) phase was detected, with no presence of secondary phase. By comparing XRD data with standard JCPDS no. 44-0093, the synthesized powders had the same characteristic peaks as tetragonal perovskite BST. Figure 1B shows a scanning electron microscopy (SEM) image of the BST particles, where it can be seen that the particles prepared by the solid-state reaction with an equi-axed morphology exhibited a particle size of 0.2–1.2 μm. Figure 1C shows the ferroelectric polarization–electric field (*P-E*) hysteresis loops of a sintered BST pellet with the relative density of 98.9% at different temperatures ranging from 25 to 42°C; a sintered sample was simply used to facilitate characterization of the *P-E* although powders will be used for piezo-catalysis. At room temperature, the saturated polarization and coercive field were ∼3.5 μC cm^−2^ and ∼1 kV cm^−1^, respectively. With an increase of temperature, the polarization decreased and the hysteresis loops became slimmer and the hysteresis loop exhibited an almost linear response at 42°C, which can be regarded as the Curie temperature, similar to Ashish's observation (Pradeep et al., 2010). This is also in accordance with the dielectric properties illustrated in Figure 1D, where the relative permittivity (*ε*) is found to initially increase with temperature, and then reaches a maximum at the Curie temperature of *T*_*c*_ ∼ 42°C, and is then followed by a decrease in permittivity with increasing temperature that can be assigned to the transition from the asymmetric tetragonal (ferroelectric) to symmetric cubic (paraelectric) phase. The dielectric loss (loss tangent) exhibited a similar trend but remained low in the temperature range of 26.5–65.5°C. The AC conductivity (*σ*) increased with frequency *f*, based on the equation of *σ* = *2πfε*, and there is only a limited change in AC conductivity in temperature range of 25–42°C, as shown in Figure S1. Figure 1E shows the domain pattern and the local surface displacement via piezo-force microscopy (PFM) as a function of the DC bias voltage from −10 V to 10 V. As shown in Figure 1E-a, a well-defined hysteresis response of the BST sample can be observed locally, further confirming the ferroelectricity in BST ceramic below its Curie temperature. The local surface displacement due to the converse piezoelectric effect is shown in Figure 1E-b, indicating the magnitude of the piezoelectric coefficient along the normal direction with a mean d33∗ of ∼430 pm V^−1^, calculated from the PFM amplitude. An approximately 180° phase difference (from ∼−20° to ∼160°) can be found in Figure 1E-a, together with two different color contrasts with an ∼180° phase difference, shown in Figure 1E-c, indicating the switching nature of the domains in the BST material. In summary, the material is clearly ferroelectric and exhibits a low Curie temperature of *T*_*c*_ ∼ 42°C, which makes BST a suitable candidate for the investigation of the effect of working temperature on piezoelectrically enhanced water splitting.

We now examine the influence of working temperature on the water splitting capability of BST, where the experimental setup is outlined in Figures S2 and S3. The apparatus was used to apply ultrasound to BST powder dispersed in a mixture of water and methanol, which was used as a sacrificial reagent to scavenge OH radicals (Penconi et al., 2015). As can be seen in the Figure S4, the application of ultrasound at a fixed power level and frequency (40 kHz) leads to an increase in temperature of the bulk solution with excitation time. Because sono-chemical hydrogen production can be affected by ultrasonic frequency, dissolved gas, acoustic power, and liquid temperature (Rashwan et al., 2019), they were closely controlled to ensure a stable sono-chemical hydrogen generation, as in Figure S4, to enable analysis of the effect of working temperature on piezo-catalytic hydrogen production. To select a specific working temperature, without altering other conditions, the hydrogen production rates at the same ultrasound *on*/*off* time ratio (1/1) with different interval times were determined using initial water bath temperatures of 23, 30, and 33°C. This ensured that the ultrasound was applied in a narrow working temperature range in relation to the *T*_*c*_ of the material. In order to separate the amount of hydrogen that was generated by the ultrasound *on* and *off* processes, the product gases during *on* and *off* time periods were continuously swept by an argon stream and sent directly to the gas chromatography for immediate hydrogen measurement, as in Figure S2. When the ultrasound *off* time was greater than the retention time of the gases in the system, any hydrogen produced was fully swept out of the measurement system; when the *off* time is shorter than the retention time background hydrogen is detected due to accumulated hydrogen in the system. Measurement uncertainties have been estimated by at least three experiments.

**Figure 2:**
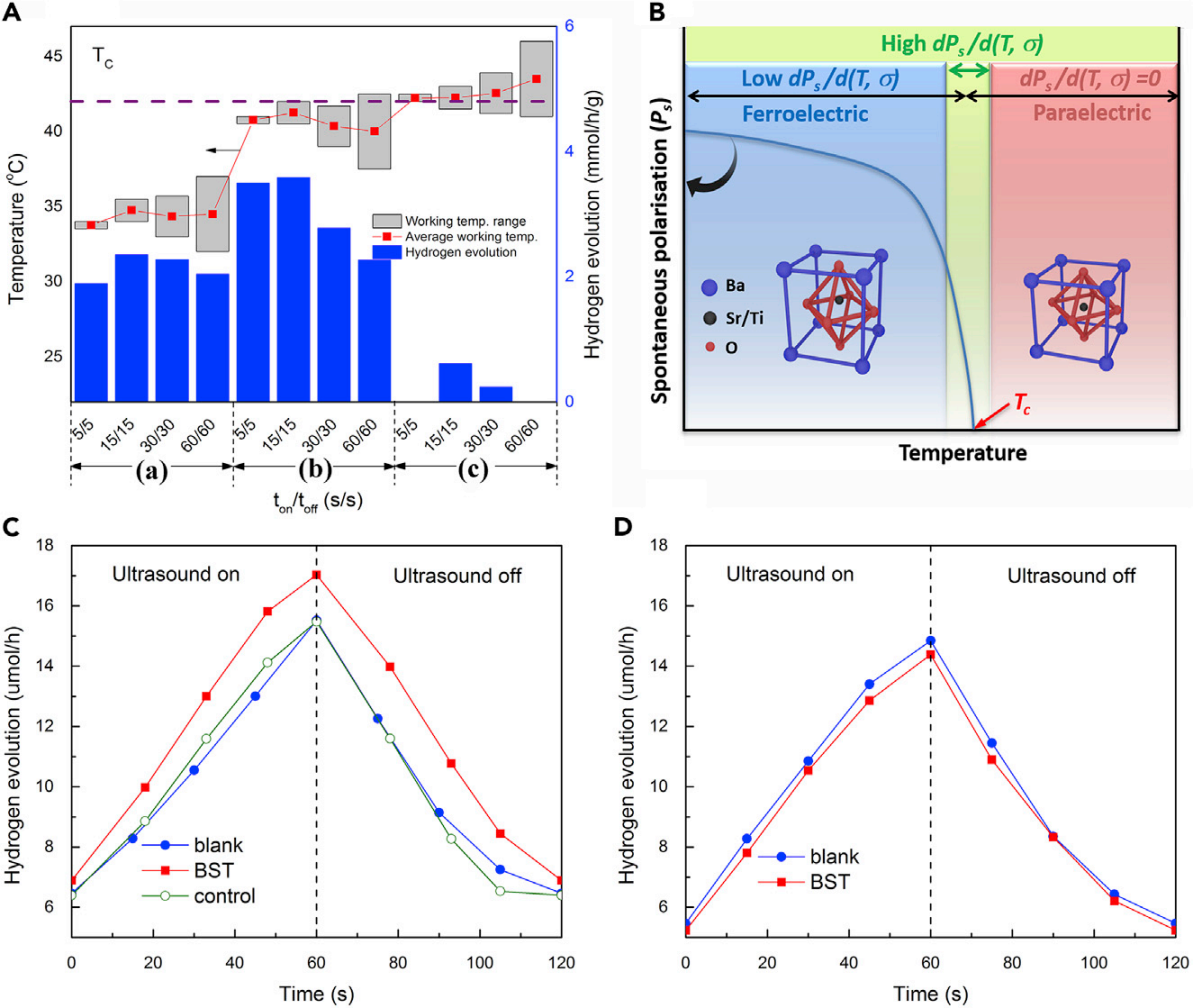
Hydrogen Evaluation under Different Water Bath Temperatures (A) Working temperature and average piezo-catalytic hydrogen generation obtained with different ultrasound on/off time ratios (t_on_/t_off_) at different water bath temperature (T_bath_), where (A), (B), and (C) relate to water bath temperatures of 23, 30, and 33°C respectively. (B) Effect of *T*_*c*_ on polarization. Total hydrogen evolution during one cycle of ultrasound excitation at different bulk solution temperature, (C) ~ 40°C and (D) ~ 43°C. Measurement uncertainties have been estimated by at least three experiments.

Figure 2A shows the working temperature and average piezo-catalytic hydrogen generation obtained with different ultrasound on/off time ratios at different water bath temperatures. It can be seen that the degree of hydrogen evolution increases as the working temperature approaches the *T*_*c*_ of the material; however, when the working temperature exceeds the *T*_*c*_ the degree of hydrogen evolution decreases because the BST is no longer ferroelectric. In addition, the highest and most distinct hydrogen evolution was obtained at temperatures of 40–42°C, near the *T*_*c*_, where the material exhibits the highest and most distinct piezoelectric coefficient. This can be explained by examination of Figure 2B (Justin Raj et al., 2011, Whatmore, 2017), which shows a schematic of the temperature dependence of spontaneous polarization of BST and its dependence with stress (piezo-coefficient). At low temperatures, below the *T*_*c*_, the piezo-coefficients of the ferroelectric tetragonal phase are relatively small, due to the relatively small changes in polarization in response to a stress. As the temperature approaches *T*_*c*_, greater changes in polarization are expected with an applied stress change and the piezo-coefficients increase, leading to an increase in piezo-catalytic performance due to the application of ultrasound. Above the *T*_*c*_ the material changes to the paraelectric cubic phase and exhibits no change in polarization with ultrasound.

Figure 2C shows that use of inert α-Al_2_O_3_ (as a non-ferroelectric control) did not alter the hydrogen evolution, whereas the addition of ferroelectric BST resulted in a large difference compared with both “control” (α-Al_2_O_3_) and “blank” (no addition of any solid substances) tests. In addition, further evidence of no enhancement in hydrogen production at a working temperature that exceeds the *T*_*c*_ is seen in Figure 2D, indicating that a loss in the polarization of the material (see Figures 1C and 1D) correlates with a reduction in hydrogen production. Clearly, there is a strong link between the temperature change, working temperature, and the hydrogen production in the presence of BST, whereas the control and blank test is not influenced, confirming again the piezo-catalytic activity of BST. The dispersion of BST particles in an aqueous solution and powder microstructure morphology after ultrasound excitation are shown in Figure S5, which indicate the well-dispersed state of BST particles in the solution and no significant change in morphology due the application of ultrasound. The fact that the efficiency of the chemical reaction was reduced when the working temperature was higher than the *T*_*c*_ indicates that any increased thermal motion of the dispersed powder due to the increased temperature was not the primary reason for the improved catalytic performance (Mushtaq et al., 2019).

**Figure 3:**
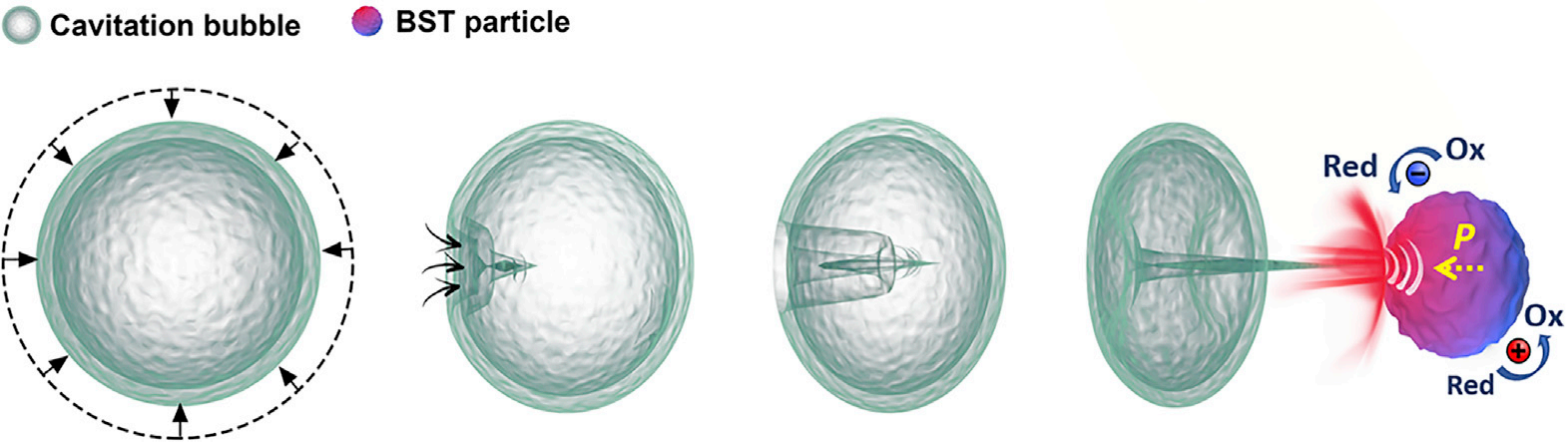
Schematic of the Effect of Cavitation on a BST Ferroelectric Particle under the Application of Ultrasound The compressive shock wave acts to reduce the level of polarization, leading to the formation of surface charges for electrochemical reaction, such as H_2_ generation.

Figure 3 shows the proposed mechanism of the generation of surface charge induced by the application of ultrasound and generation of cavitation bubbles. In an ultrasonic bath, cavitation bubbles (Haar, 2015) can be formed as a result of the high-intensity ultrasound, where the cavitation bubbles expand during the rarefaction and collapse in the compression of the sound wave; see also Figure S6. A shock wave or microjet is produced from the collapsed bubbles, which leads to a high temperature and pressure (Kenneth andSuslick, 1998) with intense local heating of 5000 K, large pressures of 500 atm, heating/cooling rates of > 10^9^ K sec^−1^, and liquid jet streams of 360 km h^−1^ (Suslick, 1990). However, in the presence of small collapsed water bubbles, typically 10–20 μm in size (Xu et al., 2015), the temperature of BST particles are likely to rapidly fall to the working temperature of the water bath and monitored in real time. Therefore, it is believed that the BST particles in the ultrasonic bath can be subjected to a compressive load (*σ*) normal or perpendicular to the polarization direction of a ferroelectric domain, which can act to change the polarization of the BST particles, and this has a greater effect near the *T*_*c*_. It is of interest to consider the differences in the generated voltage and charge as the temperature increases up to the Curie temperature. The voltage (*V*) is determined by the piezoelectric voltage coefficient, *g*_*ij*_ *= d*_*ij*_*/ε*, and is a measure of electric field per unit stress; the *g*_*ij*_ coefficient decreases with temperature as a result of an increase in permittivity. The level of charge (*Q*) is determined by the piezoelectric charge coefficient, *d*_*ij*_*,* which reaches a maximum at its *T*_*c*_ (Miclea et al., 2007, Chen et al., 2014). Based on Faraday's law, *m* = *QM*/*Fz*, where *m* is the mass of H_2_, *Q* is the total electric charge, *F* = 96.485 C mol^−1^ is the Faraday constant, *M* is the molar mass of the substance, and *z* is the valence number of ions of the substance; the amount of H_2_ and RhB degradation product depend on the charge generated, whereas the threshold voltage is the driving force for the chemical reactions. The large change of polarization with stress and high *d*_*ij*_ near the *T*_*c*_ is therefore beneficial to increase the quantity of charge and H_2_ generated. The overall mechanism generates a positive and negative charge on opposing surfaces (Equation 1) (Xu et al., 2018b).

(σ)

(Equation 1) BST → BST + *q*^*-*^ + *q*^*+*^

The positive pyroelectric charges (*q+*) are able to oxidize water molecules at the surface of the BST particle, thereby producing H^+^ and O_2_ (Equation 2):

(Equation 2) *2q*^*+*^ + H_2_O → 2H^+^ + ½O_2_

The H^+^ can react with the negative pyroelectric charges (*q*^*-*^) to create hydrogen (Equation 3).

(Equation 3) 2H^+^ + 2*q*^*-*^ → H_2_

Above the *T*_*c*_ the polarization is lost and no pyroelectric charges are produced (Equation 1) and the piezo-catalytic activity is therefore reduced, as in Figures 2A and 2D.

**Figure 4:**
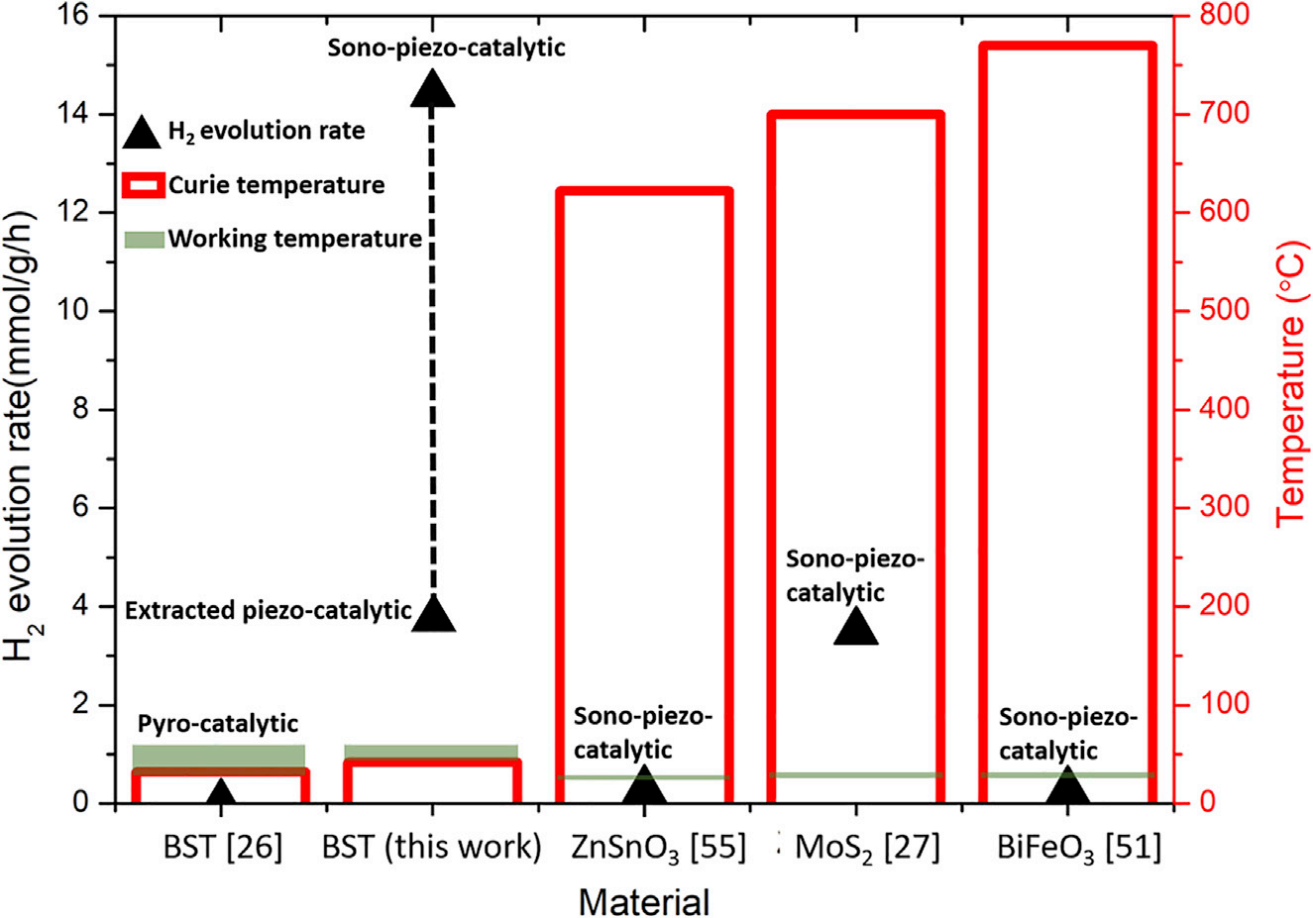
Comparisons of Hydrogen Evolution Rate under Different Conditions

Figure 4 and Table S1 provide a summary of the hydrogen evolution rates using different methods reported in the literature. It is clear that by optimization of the working temperature in relation to the *T*_*c*_ a promising piezo-catalytic hydrogen production rate of 3.59 mmol/g/h is achieved, which is much larger than those obtained from a simple pyro-catalytic effect from BST (7.8 μmol/g/h) (Xu et al., 2018b) and from other piezoelectric materials (Wang and Wu, 2020, You et al., 2019, Su et al., 2018), with a Curie temperature of 32°C (Ba_0.7_Sr_0.3_TiO_3_) (Xu et al., 2018b), 820°C (BiFeO_3_) (Kiselevet al., 1963), 622°C (MoS_2_) (Xia et al., 2016), and >700°C (ZnSnO_3_) (Zhang et al., 2010). The production rate also remained stable for a testing period of up to 700min, see Figure S7.

**Figure 5:**
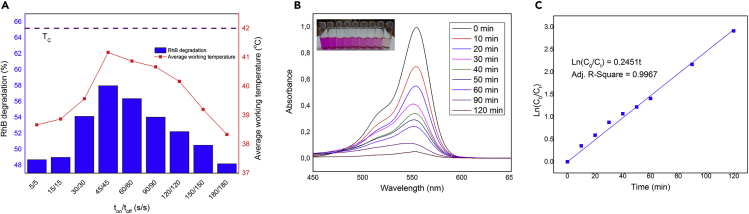
RhB Degradation Performance under Different Water Bath Temperatures (A) Average working temperature and RhB degradation obtained after 30 min of excitation time with different ultrasound *on*/*off* time ratios. (B) Absorbance spectra of RhB solution after different treated time at average working temperature of 41°C with *on*/*off* ratio of 45s/45s in the presence of BST. (C) Kinetic plot of *ln*(*C*_*0*_/*C*_*t*_) vs irradiation time for RhB degradation.

A similar sensitivity of performance in relation to *T*_*c*_ is also observed in the case of sono-piezo-catalytic degradation of RhB, as can be seen in Figure 5A. The highest working temperature was observed in the case of ultrasound with a 45 s on/off interval, 41°C, respectively, exhibiting the highest RhB degradation of 58%. The degradation rate of RhB then decreased with an increase in the time interval due to decrease in working temperature. Figure 5B shows that the characteristic adsorption peak of RhB gradually decreased with increasing reaction time and complete degradation of the RhB could be achieved after 120 min. From the above results, it can be concluded that approaching *T*_*c*_ is the most important strategy that should be considered for maximizing the pyro-catalytic effect. Figure 5C illustrates the kinetics of the RhB degradation, where a linear relationship between ln(*C*_0_/*C*_*t*_) and irradiation time for the degradation of RhB can be fitted to ln(*C*_0_/*C*_*t*_) = *kt*, where *C*_*0*_ and *C*_*t*_ are the RhB concentration at time *t*_*0*_ and *t* respectively, and *k* is the apparent first-order rate constant (min^−1^). The slope of the straight line represents the kinetic constant *k* with a calculated value of 0.0245 min^−1^ and R^2^ of 0.9967, implying a good catalytic activity that is comparable to that of commercial Degussa P25 for sono-catalytic degradation of RhB (Zhou et al., 2015).

### Conclusion

In summary we have provided the first experimental demonstration of the importance of working temperature, in relation to the Curie temperature for piezo-catalytic hydrogen generation and degradation of water contaminants (RhB) under ultrasound. The piezo-catalytic performance of the ferroelectric materials increases significantly near the *T*_*c*_ due to an increase in piezo-coefficients and the impact of cavitation of the local stress; further work to examine any potential pyroelectric contributions due to the rapid localized temperature changes at cavitation sites would be of interest. A wide variety of lead-free ferroelectrics are currently under investigation (Zheng et al., 2018), and although the low *T*_*c*_ of particular systems makes them inappropriate for conventional sensor or actuator applications, they would be of interest for such piezo-catalysis. Under optimum conditions of operation near *T*_*c*_ the addition methanol at only 4% can enhance the hydrogen evolution with negligible generation of other impurities. Consequently, a high hydrogen production rate of 10.83 μmol/h was achieved, which is much higher than previously reported, such as 3.4 μmol/h by Wang et al. (Wang et al., 2010) (see Table S1). We also demonstrate the need for careful control of experiments to separate both sono-chemical and pyro-chemical effects. It is also of interest to explore other modes of operation such as piezo-photo-catalytic effects (Tan et al., 2015, Huang et al., 2017) near *T*_*c*_ or mixing ferroelectric particles of different *T*_*c*_ to enable operation over a range of temperatures. Of particular interest here is that for the materials selected for this study, Ba_x_Sr_1-x_TiO_3_, the Curie temperature can be tailored from −200°C to 120°C (Airimioaei et al., 2017, Mbarki et al., 2014) and provides ample opportunities to tune the Curie temperature for a range of working temperatures for optimum piezo- and pyro-catalysis.

### Limitations of the Study

Particles of ferroelectric were suspended in water in this work, and methods to disperse larger amounts of particulates in water are needed to improve the amount hydrogen formed or allow scale-up for water treatment. Potential avenues of research could include porous materials. Although ultrasound was used in this work, harvesting ambient sources of energy would be beneficial; this could include inducing cavitation from water flow or using temperature fluctuations.

## Methods

All methods can be found in the accompanying Transparent Methods supplemental file.

## Data and Code Availability

The datasets generated during and/or analyzed during the current study are available from the corresponding author on reasonable request.

## Supplemental Information

Documents S1. Transparent Methods, Figures S1–S7, and Table S1
