# Supplementary material for: Demonstration of Enhanced Piezo-Catalysis for Hydrogen Generation and Water Treatment at the Ferroelectric Curie Temperature
Source: iScience. 2020 Apr 24;23(5):101095. doi: 10.1016/j.isci.2020.101095 (PMC7215196; doi:10.1016/j.isci.2020.101095)
Supplement: Documents S1. Transparent Methods, Figures S1–S7, and Table S1 [file mmc1.pdf]

iScience, Volume *23*

Supplemental Information

Demonstration of Enhanced Piezo-Catalysis

for Hydrogen Generation and Water Treatment

at the Ferroelectric Curie Temperature

Pham Thi, Thuy Phuong, Yan, Zhang, Nick, Gathercole, Hamideh, Khanbareh, Nguyen Phuc, Hoang Duy, Xuefan, Zhou, Dou, Zhang, Kechao, Zhou, Steve, Dunn, and Chris, Bowen

Supporting Information

Transparent Methods

Material preparation:

$Ba_{0.75}Sr_{0.25}TiO_3$ (BST) powders were prepared by a solid-state reaction. Analytical grade (Sigma Aldrich) barium carbonate ($BaCO_3$, 99%), strontium carbonate ($SrCO_3$, 99%), and titanium dioxide (TiO_2, 99.9%) were selected as starting materials and weighed according to their stoichiometric ratio. The above mixtures were calcined at 1200 °C for 3 h, followed by additional ball-milling for 24 h. The milled powders were mixed with 1 wt.% poly(vinyl alcohol) (PVA) binder and dried in an oven at 60 °C. To form dense materials for characterisation, the powders were uni-axially cold-compacted to form pellets of 10 mm in diameter and 1 mm in thickness. The pellets were first heated to 500 °C for 3h to remove the binder and then sintered at 1325 °C for 4 h.

Characterization:

The phase structure of the BST powders was examined by X-ray diffractometer (BRUKER D8-Advance, USA) with Cu radiation with 2θ ranging from 20°-70°. The morphology of the BST powders was examined by a scanning electron microscopy (SEM, JSM6480LV, Tokyo, Japan). For electro-physical measurements of sintered materials, silver paint was coated on both working faces of the sintered samples to form electrodes. The temperature dependence of the dielectric constant (relative permittivity) and polarization of the unpoled pellets were studied in a temperature range of 26-67 °C using an impedance analyzer (Solartron 1260, Hampshire, UK) at a frequency of 1 kHz and a Radiant RT66B-HVi Ferroelectric Test system at hysteresis period of 10 ms, respectively. Piezo force microscopy (PFM) in contact mode of an atomic force microscope (NanoManTM VS) with a conductive Pt/Ir-coated Si cantilever (SCM-PIT) was used to investigate the piezo-response phase and amplitude of BST sample. The ceramic sample was polished, thermally etched, and ground to 0.2 mm before measurement. A direct current (DC)

voltage from -10 to 10V was applied superimposed on an AC modulation voltage during polarisation switching.

Hydrogen production experiments:

A mass of 1 mg of BST powder was added and dispersed into a 20 mL headspace vial containing 10 mL of distilled water and methanol (Merk) mixture. After sealing tightly, it was placed in the center of an ultrasonic cleaner bath (60W-TP01-Taiwan Total Meter) and purged with argon for 15 min to completely remove air prior to being sonicated for a desired reaction time at a frequency of 40 kHz (see **Figure S2**). For online measurement, the generated hydrogen was continuously swept by argon stream at about 12 ml/min and passed to a 120 mL-condensation bottle before sending to a gas chromatograph (HP 5890 Series II, Agilent) equipped with a thermal conductivity detector and RT-Msieve 13X capillary column (30 m x 0.32 mm, Thames Restek) by ChemStation Software (see Figure S3). Actual flow rates were verified with a soap-film bubble flowmeter (Hewlett-Packard) prior to each run. The hydrogen production rate was calculated using a calibration curve that was made by using different concentrations of hydrogen diluted in argon. During the experiment, the ultrasonic bath temperature was controlled by circulating cooled water back to the ultrasonic bath. The *control* (non-ferroelectric particulate addition) tests were performed at the same conditions with 1 mg of α-Al_2O_3 (Merk) as an inert material and *blank* tests without adding any solid particle to the reaction mixture. This allowed the *sono-chemical* contribution to hydrogen generation be determined. The uncertainties have been estimated by at least three experiments. The *piezo-catalytic* hydrogen generation was calculated by simple subtraction of hydrogen evolution rates obtained from experiments with BST and blank tests due to no statistical difference in hydrogen generated from control and blank tests, then divided by the amount of BST.

Rhodamine B degradation experiments:

A mass of 2 mg of BST powder was added and dispersed into a 20 mL headspace vial containing 10 mL of RhB solution (10 mg/L), which has been widely chosen as a model contaminant based on its popularity among research groups (Rochkind et al., 2015). By conducting mutually exclusive experiments without

applying ultrasound, it was found that the direct photolytic degradation, adsorption and thermal degradation of RhB on BST are negligible. Thus, all further experiments were performed at normal light condition and without adsorption pre-treatment. The absorption spectra of the treated samples were acquired with a UV-Vis spectrophotometer.

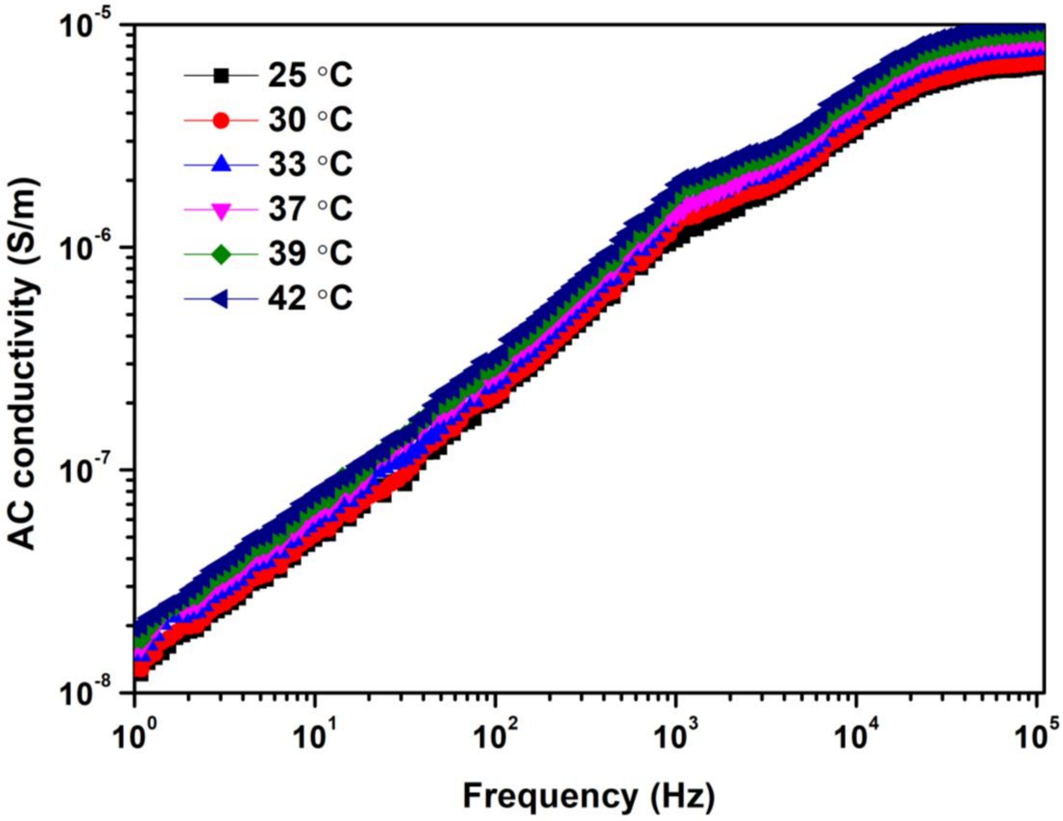

Figure S1 AC conductivity of BST with different frequencies and temperatures, related to Figure 1.

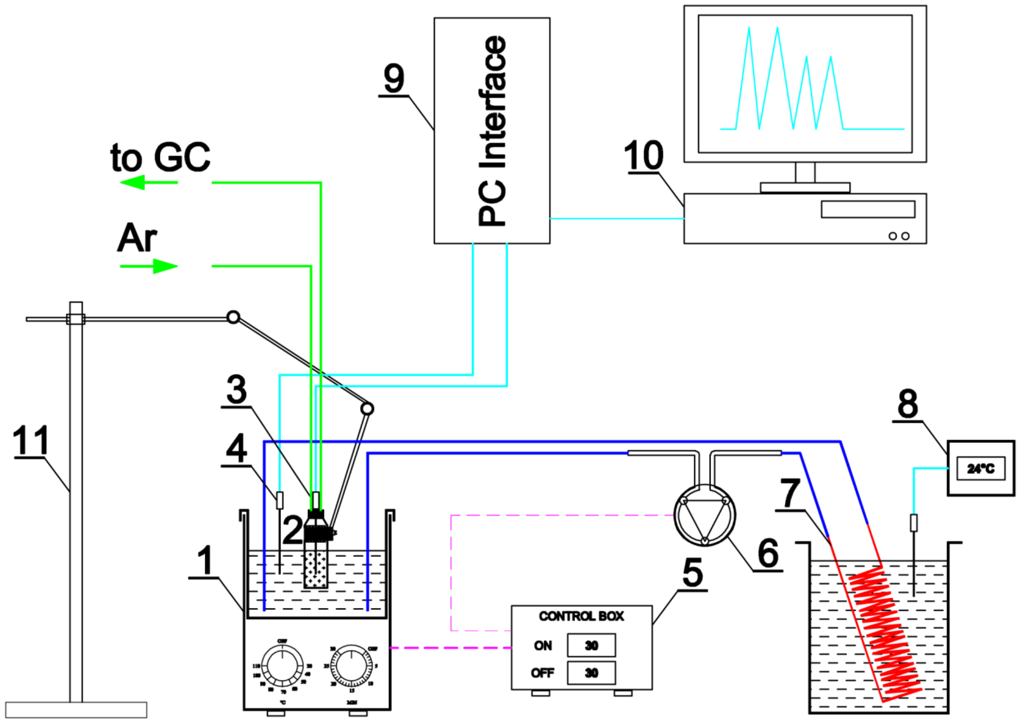

Figure S2. Schematic image of the experimental setup. 1 – Ultrasonic bath; 2 – Sample vial; 3 – Inside temperature sensor; 4 – Water bath temperature sensor; 5 – Timers; 6 – Peristaltic pump; 7 – Cooling coil; 8 – Cooling bath temperature sensor; 9 – Interface; 10 – Computer; 11 – Flexible stand, related to Figure 2.

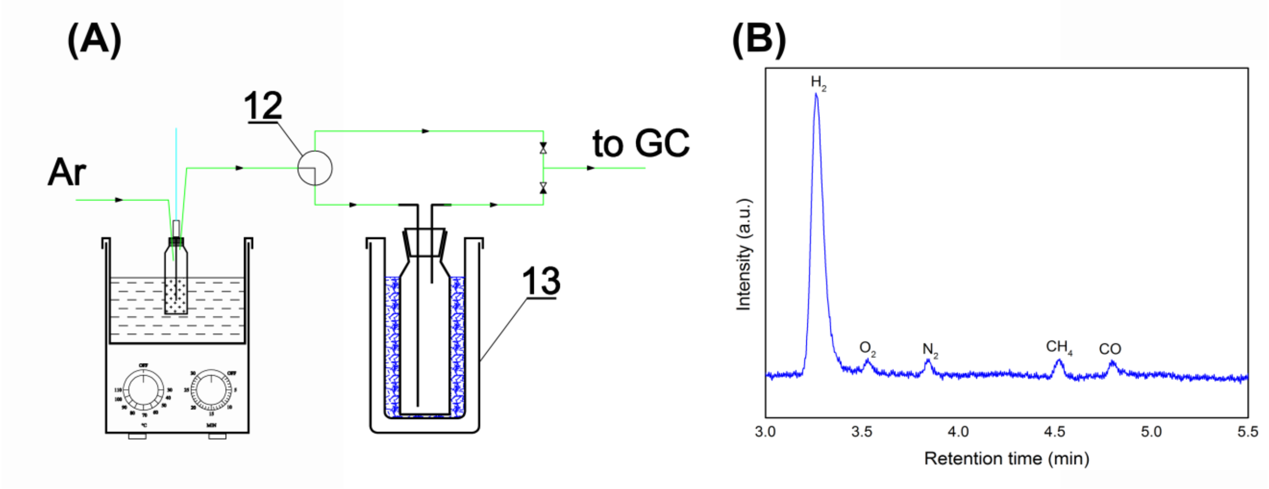

Figure S3. (A) Online hydrogen measurement setup. (B) Representative GC-TCD chromatogram of the produced sample at optimum conditions. 12 – Three ways valve; 13 – Condensation bottle, related to Figure 2.

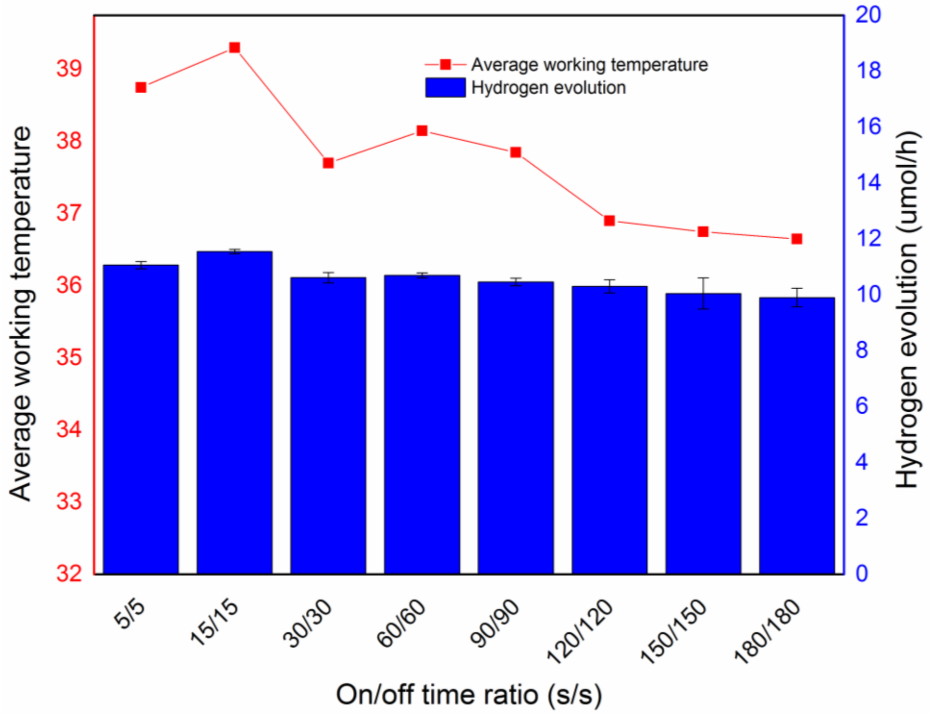

Figure S4. Hydrogen generation for blank test, with sono-chemcial produced hydrogen shown to be temperature independent, related to Figure 2.

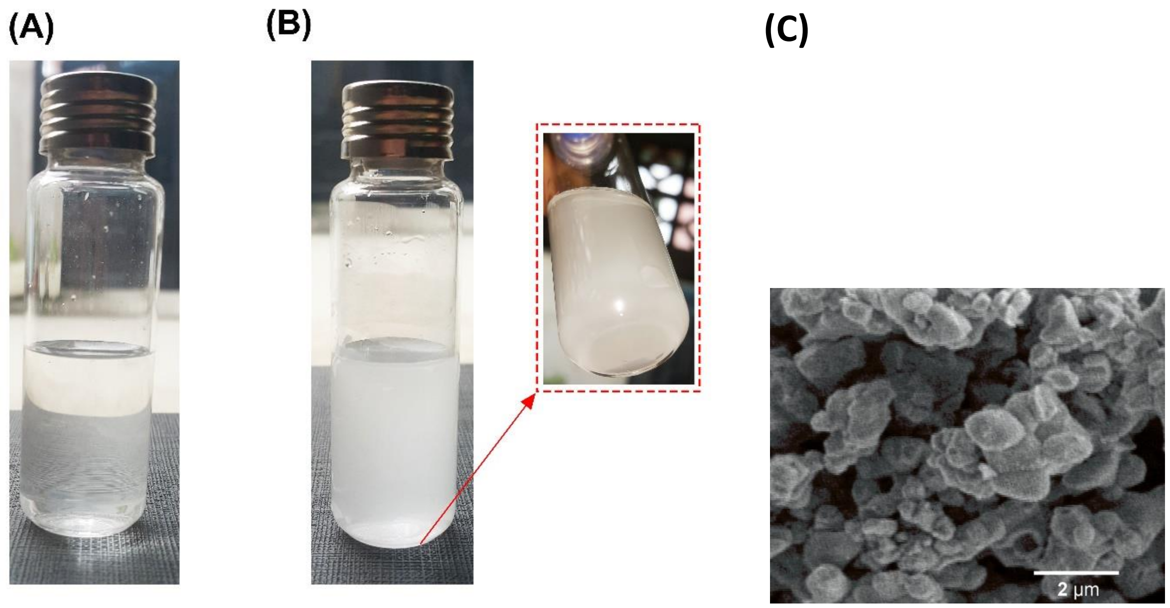

Figure S5. Images of the aqueous solution before and after powder addition. (A) pure water, (B) BST aqueous suspension after the application of ultrasound excitation, inset shows no precipitation can be seen on the bottom side, (C) SEM image of the BST powder after the application of ultrasound, related to Figure 2.

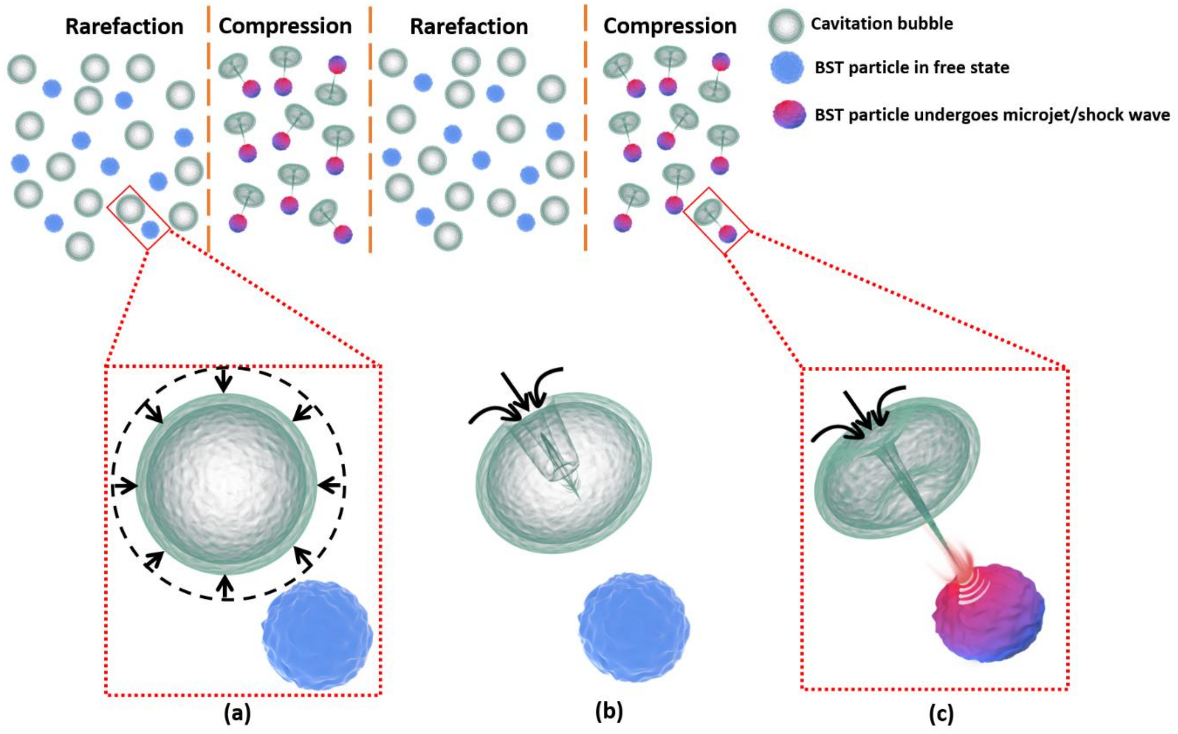

Figure S6. Schematic of ultrasound applied to ferroelectric particle, related to Figure 3.

Table S1 Rate of hydrogen evolution under different conditions, , related to Figure 4.

**Effect**	Material	**Hydrogen evolution rate**		**Reference**
		µmol/h	mmol/h/g	
Pyro-catalytic	BST	n/a	0.0078 [a]	(Xu et al., 2018)
Sono-chemical		3.4 [b]	n/a	(Wang et al., 2010)
		10.83 ± 0.13 [c]	n/a	This work
Sono-catalytic	*Au/TiO₂*	282.3 [b]	3.8 [b]	(Wang et al., 2010)
Piezo-photo-catalytic	$KNbO_3/MoS_2$	n/a	0.096 [d]	(Jia et al., 2019)
Sono-piezo-catalytic	BST	14.42 ± 0.15 [c]	14.42 ± 0.15 [c]	This work
	$ZnSnO_3$	n/a	3.5 [e]	(Wang and Wu, 2019)
	MoS_2	n/a	0.028 [f]	(Su et al., 2018)
	$BiFeO_3$	n/a	0.026 [g]	(You et al., 2019)
Extracted piezo-catalytic effect	BST	3.59 [c]	3.59 [c]	This work

n/a abbreviates either not available or not applicable

[a] *Thermal cycle is 10 min with thermal fluctuation of 25-50 °C, concentration of methanol is 20 vol%*

[b] *Concentration of methanol was 4 vol%, ultrasound source of 40kHz at 50 W*

[c] *Concentration of methanol was 4 vol%, ultrasound source of 40kHz at 60 W, ultrasound on/off time ratio is 15/15s with thermal fluctuation of 40.5 - 42 °C*

[d] *Triethanolamine was used as a sacrificial reagent (15%), ultrasound source of 40kHz at 110 W*

[e] *Anhydrous alcohol was used as a sacrificial reagent (50 vol%), ultrasound source of 40kHz at 250 W*

[f] *Fe2+ was used as inorganic oxidizable sacrificial agent, ultrasound source of 40kHz at 140 W*

[g] *Na_2SO_3 was used as a sacrificial reagent (0.05M), ultrasound source of 45kHz at 50 W*

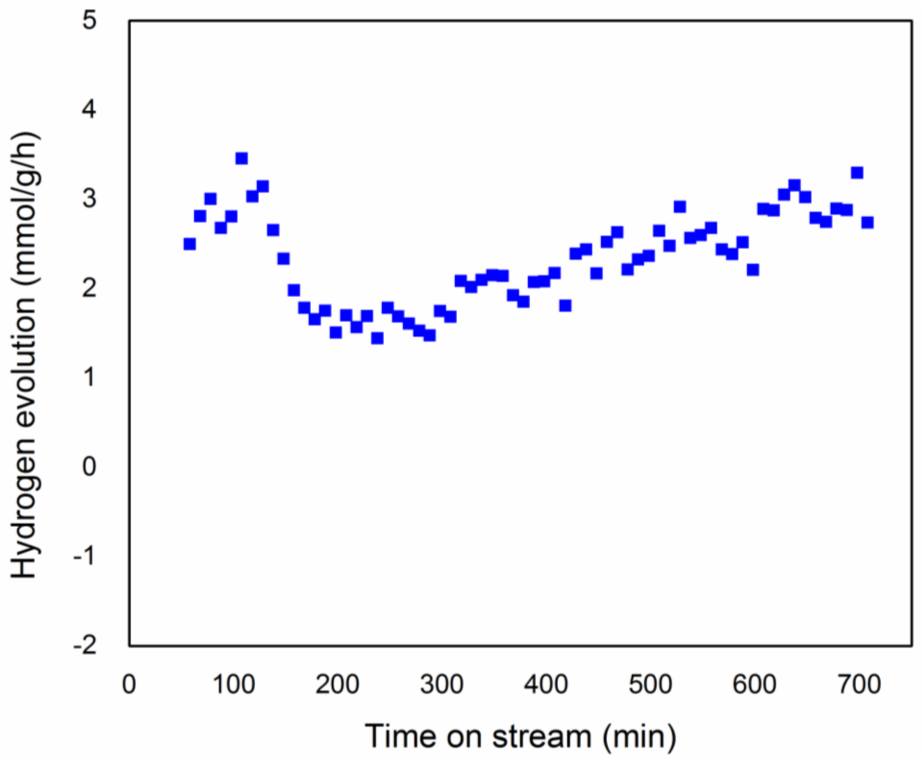

Figure S7. Piezo-catalytic hydrogen generation with time on stream, related to Figure 4.

In this experiment, a condensable bottle (see Fig. S2-A) was used to determine a stable hydrogen production rate. Thus, there is a spike at ~100 mins which is the required duration time for the H_2 to fully fill the bottle. H_2 production was found to be affected by methanol concentration, therefore a subsequent decrease (200min) may be a result of a decrease in methanol concentration due to its vaporisation. At long times, >200min the sample volume is likely to decrease during ultrasound excitation, resulting an increase in inside temperature (outside temperature and ultrasound power were constant), thus, an increase of H_2 production rate may occur for longer durations.

Reference

JIA, S., SU, Y., ZHANG, B.-P., ZHAO, Z., LI, S., ZHANG, Y., LI, P., XU, M. & REN, R. 2019. Few-Layer MoS_2 Nanosheet-Coated $KNbO_3$ Nanowire Heterostructures: Piezo-Photocatalytic Effect Enhanced Hydrogen Production and Organic Pollutant Degradation. *Nanoscale*.

ROCHKIND, M., PASTERNAK, S. & PAZ, Y. 2015. Using Dyes for Evaluating Photocatalytic Properties: A Critical Review. *Molecules,* 20**,** 88-110.

SU, Y., ZHANG, L., WANG, W., LI, X., ZHANG, Y. & SHAO, D. 2018. Enhanced H_2 evolution based on ultrasound-assisted piezo-catalysis of modified MoS_2. *Journal of Materials Chemistry A,* 6**,** 11909-11915.

WANG, Y.-C. & WU, J. M. 2019. Effect of Controlled Oxygen Vacancy on H2-Production through the Piezocatalysis and Piezophototronics of Ferroelectric R3C $ZnSnO_3$ Nanowires. *Advanced Functional Materials,* n/a**,** 1907619.

WANG, Y., ZHAO, D., JI, H., LIU, G., CHEN, C., MA, W., ZHU, H. & ZHAO, J. 2010. Sonochemical Hydrogen Production Efficiently Catalyzed by Au/TiO_2. *The Journal of Physical Chemistry C,* 114**,** 17728-17733.

XU, X., XIAO, L., JIA, Y., WU, Z., WANG, F., WANG, Y., HAUGEN, N. O. & HUANG, H. 2018. Pyro-catalytic hydrogen evolution by $Ba_{0.7}Sr_{0.3}TiO_3$ nanoparticles: harvesting cold–hot alternation energy near room-temperature. *Energy & Environmental Science,* 11**,** 2198-2207.

YOU, H., WU, Z., ZHANG, L., YING, Y., LIU, Y., FEI, L., CHEN, X., JIA, Y., WANG, Y., WANG, F., JU, S., QIAO, J., LAM, C.-H. & HUANG, H. 2019. Harvesting the Vibration Energy of $BiFeO_3$ Nanosheets for Hydrogen Evolution. *Angewandte Chemie International Edition,* 58**,** 11779-11784.
